# Gingerols synergize with anthocyanins to induce antioxidant activity *in vitro*

**DOI:** 10.3389/fnut.2023.1229015

**Published:** 2023-09-08

**Authors:** Amna Emhemed Abdurrahim, Vera C. Mazurak, Lingyun Chen

**Affiliations:** ^1^Department of Agricultural, Food and Nutritional Science, University of Alberta, Edmonton, AB, Canada; ^2^Department of Food and Nutritional Science, College of Medical Technology-Misurata, Misurata, Libya

**Keywords:** anthocyanins, cellular antioxidant activity, cytoprotective effect, gingerols, oxidative stress, synergistic effect

## Abstract

Oxidative stress caused by free radicals contributes to the pathogenesis of multiple chronic health conditions. Phytochemicals protect against oxidative stress; however, low bioavailability from dietary sources limits their health benefits. This study aimed to assess the effects of anthocyanins and gingerols’ combination on the cellular antioxidant response of Caco-2 cells against oxidative stress. A strong synergism was observed for anthocyanin-gingerol (Ac-G) w/w combined ratios of 8:1 and 2:1 (dosages of (1 + 0.125) and (1 + 0.5) μg/mL) in the cellular antioxidant activity (CAA) and cytoprotective effects, with synergistic effect indicator (SE) values of 1.41 and 1.61, respectively. The synergism of Ac-G combinations promoted cellular antioxidant defense systems and cytoprotective effects by reducing the induced GPx enzyme activity, protecting SOD enzyme activity, reducing cellular ROS generation, increasing glutathione content, and inhibiting lipid peroxidation. Thus, Ac-G combinations showed potential in supporting the endogenous antioxidant systems to protect cells from oxidation and restore physiological redox status. The Ac-G formulation is a promising healthy option that can be developed into functional foods or nutraceutical products. Furthermore, it could help address the low bioavailability of these phenolics, as higher effects were achieved when combining the same doses.

## 1. Introduction

Oxidative stress induced by free radicals has been implicated in the pathogenesis of multiple chronic health conditions such as atherosclerosis, cancer, neurodegenerative, and coronary heart disease (1). Reactive oxygen species (ROS) formed in the cells of aerobic organisms and the primary free radicals can damage biological systems. They are generated endogenously as by-products of mitochondrial-catalyzed electron transport reactions and inflammatory cell activation processes. However, free radicals can also be produced exogenously by environmental agents (2). It has been established that ROS have physiological roles in many cellular signaling systems, such as the cellular responses in the defense against infectious agents and exerting control over redox-regulated signaling. However, high production of ROS can be harmful by damaging and causing dysfunction of some cellular molecules and structures, which may contribute to disease processes (2, 3).

Antioxidant capacity in the body is a complex defense network that includes powerful oxidative stress response systems. These consist of endogenous systems, enzymatic and non-enzymatic, which act together at different levels to induce free radical prevention and scavenging and radical-induced damage repair (1). For example, the cellular antioxidant enzymatic system includes several enzymes that convert free radicals into non-radical or less reactive species. Glutathione peroxidase (GPx) is a family of enzymes in different cellular components that play an essential role in detoxifying hydroperoxides through the glutathione (GSH) system (4).

The GSH system is essential in maintaining the intracellular redox status. It is a tripeptide thiol and has two forms: reduced (GSH) and oxidized (GSSG) forms, and its protective action against ROS is facilitated by two antioxidant enzymes, GPx and glutathione reductase (GR) (5). GSH is typically produced and recycled in the body, but oxidative stress can suppress this mechanism and deplete GSH. GSH depletion can be caused by factors such as diet, medications, stress, infections, and pollution (6).

The imbalance between antioxidant protective defenses and free radicals causes oxidative stress and damages protein, lipid, and DNA structures. Natural antioxidants have been used to mitigate oxidative stress as they support the antioxidant defenses to limit and prevent the toxic effects of free radicals (7). Phytochemicals from fruits, vegetables, and herbs have the ability to scavenge ROS, protect cells against oxidative stress-induced damage, and modulate signal transduction of several signals involved in antioxidant responses to oxidative stress (8).

Anthocyanins are phenolic compounds that constitute the largest group of water-soluble pigments in fruits and vegetables. They have been reported to exert various biological functions, including anticarcinogenic and anti-inflammatory activities and preventing cardiovascular diseases (9). The antioxidant function is one of the critical mechanisms related to their health benefits; therefore, anthocyanin extracts have been applied to functional foods and natural health products (9). Naturally occurring anthocyanins show comparable antioxidant activity to synthetic antioxidants such as butylated hydroxyanisole (BHA) and butylated hydroxytoluene (BHT) (10). Still, they are safely consumed at higher doses (11). Anthocyanins have been reported to deactivate ROS, reduce DNA oxidative damage, increase glutathione content, and improve the activities of the antioxidant enzymes SOD and GPx (9, 12, 13). Dietary anthocyanins in the gastrointestinal tract have been reported to quench ROS in local cells to prevent damage to the epithelial barrier and inhibit protein expression levels of COX-2 and inflammation (14). However, the instability of anthocyanins affects their bioavailability on the one hand, and on the other, researchers reported that the metabolized products of anthocyanins could induce biological activities (15).

Ginger roots (rhizome of *Zingiber officinale* Roscoe, Zingiberaceae) have been used for thousands of years as a food seasoning and herbal medicine for health care and disease prevention. The traditional use of ginger as herbal medicine is attributed to the activity of gingerols (6-, 8-, and 10-gingerols and 6-shogaol), which are considered the main bioactive components in ginger (16). Ginger has been reported to exhibit anti-thrombotic, anti-inflammatory, anti-cancer, and anti-microbial activities, in addition to its properties in alleviating the toxicity of hepatotoxins in experimental models (16). The protective actions of gingerols are mediated through free radical scavenging and modulating the levels of detoxifying enzymes (17).

The concept of synergism between different phytochemicals in fruits and vegetables was proposed more than a decade ago (18). When a combination of two or more agents exhibits higher bioactivity or therapeutic effects than the sum effect of the individual agents at the same concentration, this effect is described as synergism. It suggests multiple cumulative or additive effects pathways as underlying mechanisms (19). Several mechanisms of action have been proposed for antioxidant synergies, such as scavenging ROS before they induce oxidative damage on cellular molecules, inhibition of oxidative enzyme activities, induction of defense enzyme expression, and modulating the expression of genes associated with redox processes (20). While most of the antioxidant synergies have been explored in chemical models, and despite their wide usage, living cells better mimic the synergy behaviors under physiological conditions (20–22).

Research suggests that, despite low plasma concentrations of anthocyanins and gingerols after oral administration, they seem to have adequate capacity to modulate signal transduction and gene expression *in vivo* to exert bioactivities in promoting health benefits (15, 23–25). Furthermore, achieving biological functions at a lower dosage of anthocyanins and gingerols by combining them might enhance their health effects. Thus, combining anthocyanins and gingerols was hypothesized to lead to a synergistic antioxidant effect against oxidative stress. Furthermore, the combined extracts exert their antioxidant effects through more antioxidant defense mechanisms than each extract alone to promote the synergistic effect. Therefore, this study aimed to investigate the combined and individual effects of anthocyanin and gingerol extracts from bilberry fruits and ginger roots, to induce cellular antioxidant activity (CAA) and cytoprotective effects as markers for cellular antioxidant capacity. Specifically, this research focused on the colorectal cancer cell model Caco-2 to study the combined antioxidant effects of anthocyanin and gingerol extracts to identify their potential synergistic interaction. The selection of the colorectal cancer cell model was based on the consideration that colorectal cancer is the third most common cancer in the word (26). In addition, anthocyanins and gingerols are mainly accumulated in the gastrointestinal tract (23, 25). Application by industry will provide a nutraceutical strategy to prevent colorectal cancer. The cellular models were focused on as a first step of this research before the animal model to reduce the practical cost of exploring synergism and for ethical considerations. Furthermore, this study investigated some of the molecular and biochemical activities of antioxidant defense mechanisms underlying the synergistic effects of combined anthocyanins and gingerols on Caco-2 cells, including ROS generation, lipid peroxidation, cellular glutathione content, and the activities of some antioxidant enzymes.

## 2. Materials and methods

### 2.1. Materials

Bilberry extract powder from bilberry (*Vaccinium myrtillus*) was purchased from Hangzhou Ningsi Biotech (Hangzhou, China). Ginger root (rhizome of *Zingiber officinale*) extract powder was obtained from Nate Biological Technology Co., Ltd. (Xi’an, China). In addition, 2′,7′-dichlorofluorescein diacetate (DCFH-DA), 2,2′-azobis (2-amidinopropane) dihydrochloride (AAPH), tert-Butyl hydroperoxide (t-BHP), monobromobimane (mBBr), 3-(4,5-dimethyl thiazolyl-2)-2,5-diphenyltetrazolium bromide (MTT), dimethyl sulfoxide (DMSO), and HPLC grade acetonitrile, methanol, and formic acid were purchased from Sigma-Aldrich Canada Ltd. (Oakville, ON, Canada). Human colon colorectal adenocarcinoma cells: Caco-2 [Caco2] (ATCC® HTB37™), 0.25% trypsin/0.53 mM EDTA in HBSS and Penicillin–Streptomycin solution (100X) were purchased from American Type Culture Collection (ATCC) (Manassas, VA, United States). Dulbecco’s Modified Eagle Medium (DMEM), DMEM/F-12 (HEPES, no phenol red), fetal bovine serum (FBS), Hanks’ Balanced Salt Solution (HBSS), and phosphate-buffered saline (PBS) were purchased from GIBCO (Burlington, ON, Canada). Diphenyl-1-pyrenylphosphine (DPPP) was purchased from Cayman Chemical (Ann Arbor, Michigan, United States). Anthocyanin standards were purchased from Polyphenols Laboratories AS (Sandnes, Norway). Gingerol standards were purchased from Sigma-Aldrich Canada Ltd. (Oakville, ON, Canada).

### 2.2. Extract analysis by high-performance liquid chromatography

The constituents of the bilberry and ginger root extracts were analyzed by reversed phase-HPLC (RP-HPLC) using an Agilent 1,200 Series HPLC System equipped with a ZORBAX Extended-C18 column (2.6 mm Å ~ 250 mm, 5 μm) and a diode array detector (G1315D) (Agilent Technologies Inc., Mississauga, ON, Canada). The chromatographic analysis of anthocyanins and gingerols in the bilberry and gingerol extracts was performed on extracts solubilized in water; after filtration of the insoluble portion, according to Yao et al. (27) and Ok and Jeong (28). The UV spectrum at 535 nm and 230 nm, respectively, were recorded. The constituents of the extracts were identified and quantified using anthocyanin and gingerol standards in concentrations ranging from 50 to 1,000 μg/mL. A standard anthocyanin mixture with known components was used as a standard for identifying the anthocyanin components in the sample corresponding to different peaks. In addition, a pure Cyanidin 3-O-glucoside standard was used to quantify anthocyanin content in the sample, expressed as mg Cyanidin 3-O-glucoside equivalent/mg extract. Individual gingerol standards were used for quantifying the gingerols. Similarly, gingerols were quantified as mg 6-gingerol equivalent/mg extract. The extracts solutions were also investigated for quantifying the water-soluble portions of the extracts, as described in Abdurrahim et al. (29).

### 2.3. Cell culture conditions and treatments

Caco-2 cells were cultured in a humidified atmosphere of 5% CO_2_ at 37°C using DMEM with 10% FBS, supplemented with 1% Penicillin–Streptomycin. The medium was changed 2 to 3 times per week. Cells were sub-cultured at the confluence. Cells were harvested by trypsinization using 0.25% trypsin/0.53 mM EDTA solution. DMEM/F-12, HEPES, without FBS, and antibiotics solution was used for the experiments.

Stock solutions (500 μg/mL) of bilberry and ginger root extracts were prepared by dissolving dry extracts in serum-free DMEM/F12 medium before being diluted. Concentrations were calculated based on the detected amounts of anthocyanins 97% and gingerols 64% from the water-soluble portions of the original bilberry and ginger root extracts, respectively (29).

First, to increase the chance of identifying synergistic effects, a wide range of anthocyanins: gingerols (Ac-G) combinations w/w (20:1-1:20) were tested in the CAA assay at 0.1–100 μg/mL doses. Combined extracts showed synergism at low doses and no significant enhancement at high doses (data are not shown). Therefore, based on preliminary trials, the amount of 1 μg/mL anthocyanins was chosen to be combined with gingerols at doses of 0.06, 0.125, 0.25, 0.5, and 1 μg/mL at Ac-G combination ratios of 16:1, 8:1, 4:1, 2:1, and 1:1, respectively, for further testing. Individual treatments at the same concentrations were applied as controls.

### 2.4. CAA assay

The CAA of anthocyanin and gingerol extracts and their combination were assessed according to the protocol developed by Wolfe and Liu (22). This assay is an *in vitro* cellular method that counts for different biological aspects such as the location of antioxidant compounds within cells, cellular uptake, and metabolism (22). Briefly, Caco-2 cells were seeded into a Black 96-well culture plate at a density of 6 × 10^4^ cells/well. After 24 h incubation at 37°C, the media was removed, and cells were washed with PBS. Then, 100 μL of fresh FBS-free medium containing the extract treatment and 25 μM DCFH-DA were added to each well, followed by incubation for 2 h at 37°C. Then, the culture medium was removed, and cells were washed with HBSS. Then, 100 μL of 600 μM AAPH in the fresh medium was applied to the cells. Positive (cells treated with oxidant and DCFH-DA but without extract treatment) and negative (cells treated only with DCFH-DA) control wells were included. Cellular fluorescence of the sample, control, and blank wells was immediately monitored each 5 min for 1 h at 37°C using a SpectraMax M3 microplate reader (Molecular Devices, San Jose, CA, United States) at emission and excitation wavelengths of 530 nm and 485 nm, respectively.

After subtracting the blank (initial fluorescence) values, the integrated area under the fluorescence curve versus time was determined for each curve. Then the *CAA* of each treatment was calculated according to Eq. (1):


(1) CAA unit%=100−∫SA/∫CA∗100

∫SA and ∫CA refer to the integrated area under the sample and positive control fluorescence curves, respectively, versus time.

### 2.5. Calculating the synergistic effect indicator of antioxidant combinations

The synergistic effects indicator (SE) was used to identify the type of interaction within the anthocyanin-gingerol (Ac-G) combinations. SE was calculated according to Fuhrman et al. (30) and Luís et al. (31). It was defined by comparing the CAA values of the combination obtained from the experiment (experimental) (EE combination) and the expected (theoretical) effect (TE combination) values, calculated as in Eq. (2):


(2) SE=EEcombination/TEcombination

where (SE > 1) indicates synergism, (SE < 1) indicates antagonism, and (SE ≅ 1) is considered additive effect. The (*TE* combination) was calculated using Eq. (3) as described by Fuhrman et al. (30).


(3) TEcombination=EE(Ac)+EE(G)−(EE(Ac)×EE(G)/100)

EE (Ac) and EE (G) are the experimental CAA values of the anthocyanin (Ac) and gingerol (G) individual extracts.

### 2.6. Cytoprotective assay

MTT assay was used to evaluate the cell viability of Caco-2 cells exposed to oxidative stress after being treated with anthocyanin and gingerol extracts and their combinations. Caco-2 cells were seeded in a 96-well plate at a density of 2 × 10^4^ cells/well and incubated for 20 h at 37°C and 5% CO_2_ humidified. The cells were pretreated with the extracts for 2 h and then washed with PBS. Cells were then exposed to 350 μM t-BHP (100 μL/well) for 24 h at 37°C. After incubation, the MTT assay was performed as previously described (29). Absorbance was measured at 570 nm. Control wells, with and without t-BHP treatment, were used as positive and negative controls, respectively. Results are expressed as a relative cell viability percentage compared to the negative control.

### 2.7. Measuring intracellular ROS

Cellular ROS generation was evaluated using the Fluorometric Intracellular ROS Kit (Sigma-Aldrich MAK143). This assay kit detects intracellular ROS by localizing fluorogenic sensors to the cytoplasm, reacting with ROS, and producing a fluorometric product. Caco-2 cells were seeded in a transparent bottom black 96-well plate at a density of 4 × 10^4^ cells/well and incubated for 20 h. After attachment, cells were incubated with the kit solution for 1 h, per the manufacturer’s instructions. Then, cells were treated with anthocyanins, gingerols, or combined extracts, and the ROS production was monitored for 3 h at 37°C using the fluorescence microplate reader. Fluorescence was assessed at excitation and emission wavelengths of 490 and 520 nm, respectively. The average fluorescence intensity after 2 h, which reflects the generated ROS, was calculated.

### 2.8. Determination of intracellular glutathione levels

Intracellular GSH levels were measured under physiological and stressed conditions using the fluorescent dye mBBr, as described by Je et al. (32) and Park et al. (33). Briefly, Caco-2 cells were seeded in a black 96-well plate at a density of 4 × 10^4^ cells/well and incubated for 24 h. First, cells were treated with individual and combined doses of anthocyanins and gingerols under normal conditions for 1 h. Control wells of cells with only the medium were included. Next, cells were washed (3x) with HBSS and labeled with 100 μL (40 μM) mBBr for 30 min at 37°C in the dark. For measurements under a stressed condition, pretreated cells were washed and exposed to t-BHP at 350 μM for 3 h, then washed and labeled as described above. The t-BHP-treated and non-treated wells were included as positive and negative controls. Then, fluorescence due to mBBr-GSH interaction was measured at excitation and emission wavelengths of 360 nm and 465 nm, respectively, every 15 min for 2 h. Results were obtained by subtracting the basal fluorescence readings of mBBr from the final readings. Therefore, the increase in fluorescence intensity indicates higher cellular content of GSH.

### 2.9. Determination of antioxidant enzymes

#### 2.9.1. Cell treatment, cell lysis, and determination of protein content

Caco-2 cells were seeded in 6-well plates at a 1×10^5^ cell/mL density and grown to the confluence. Cells were treated with extract treatments for 2 h; then, they were washed with HBSS (3×) and incubated with (150 μM) t-BHP for 24 h. Positive and negative control wells were treated and not treated with t-BHP, respectively, and they were not treated with samples. Cells were washed twice with BPS and lysed on the ice using ice-cold 300 μL of lysis solution (assay buffer/GPx assay, and 0.1 M Tris/HCl, pH 7.4 containing 0.5% Triton X-100, 5 mM β-ME, 0.1 mg/mL PMSF/SOD assay). Cell lysates were homogenized by pipetting and centrifuged (14,000 × g, 10 min, 4°C), and the supernatant was stored at −80°C and used for the antioxidant enzyme activity assays and protein content. Total protein was quantified using Pierce™ BCA Protein Assay Kit (Pierce, Thermo Fisher Scientific, Waltham, MA, United States).

#### 2.9.2. Glutathione peroxidase activity

The protective effects of anthocyanins and gingerols against oxidative stress on GPx activity were assessed. Oxidative stress was induced on the pretreated cells by t-BHP, as described above. The GPx activity was measured using an assay kit (Cat. #K762, BioVision, Inc., Milpitas, California, United States). SpectraMax M3 microplate reader was used to measure the decrease in NADPH at 340 nm, and the level is correlated to the GPx activity and presented as mU/mg protein.

#### 2.9.3. Superoxide dismutase activity

The SOD activity was evaluated using the assay kit (Cat. #K335, BioVision, Inc., Milpitas, CA, USA), which measures the total cytosolic and mitochondrial SOD activity in cell lysates. Absorbance at 450 nm was recorded using the microplate reader. SOD activity was calculated as U/mg protein using the SOD stander curve and the protein content of the cell extract.

### 2.10. Lipid peroxidation inhibitory assay

Cellular lipid hydroperoxide content was determined using the fluorescence probe DPPP, according to Bamdad et al. (34). DPPP is a non-fluorescent molecule that reacts with hydroperoxide within the cell membranes to give the fluorescent diphenyl-1-pyrenylphosphine oxide (DPPP=O)(35). Briefly, Caco-2 cells were seeded into a black 96-well microplate at a density of 5 × 10^4^ cells/well and incubated for 20 h at 37°C for attachment. First, cells were incubated with 25 μM DPPP at 37°C in the dark for 30 min. Then, they were washed with PBS (3×) and treated with anthocyanins and gingerols for 1 h. Next, cells were washed and treated with t-BHP (150 μM) for 30 min. Here, t-BHP-positive and negative controls were assigned. The fluorescence intensity of the DPPP oxide was measured using the microplate reader at excitation and emission wavelengths of 351 nm and 380 nm, respectively.

### 2.11. Statistical analysis

Data were calculated using Excel- Microsoft Office 365 software. Statistical analysis was performed using one-way ANOVA followed by the Tukey test for multiple comparisons. Differences were identified at (1) *p* < 0.05, (2) *p* < 0.01, (3) *p* < 0.005, and (4) *p* < 0.001. Statistical analysis was carried out using Origin 2020 software. Data were presented as mean ± SD (*n* = 3–5).

## 3. Results and discussion

### 3.1. Constituents of the anthocyanin and gingerol extracts

The HPLC analysis identified 15 anthocyanins (monoglycosides of cyanidin, delphinidin, malvidin, peonidin, and petunidin) in the bilberry extract (Supplementary Information 1-A). They are the most abundant anthocyanins in fruits (36). The water-soluble fraction of bilberry extract contained 97% anthocyanins by HPLC analysis (Supplementary Information 2), and the major components are delphinidin 3-O-galactoside, delphinidin 3-O-glucoside, and cyanidin 3-O-glucoside (Supplementary Information 3). The gingerols detected in the water-soluble fraction of the ginger root extract account for about 63.8% of the water-soluble fraction (Supplementary Information 2). The gingerols detected in the water-soluble fraction were 6-gingerol, 8-gingerol, 10-gingerol, and 6-shogaol (Supplementary Information 1-B), with 6-gingerol being the major component (Supplementary Information 4). The concentrations used and reported in the following sections are based on the anthocyanins and gingerols detected in the water-soluble fractions of the original extracts.

### 3.2. Effects of Ac-G combined pre-treatments on the CAA

The CAA of the anthocyanins and gingerols and their combinations in reducing the AAPH-induced ROS generation in Caco-2 cells were evaluated using the CAA assay. CAA assay is more biologically relevant than chemical antioxidant assays widely used in numerous studies. In this assay, probe DCFH-DA is cleaved and oxidized in the cells to the fluorescent dichlorofluorescein (DCF) by the oxidizing agent AAPH (22). Thus, higher fluorescence reflects higher ROS levels.

**Figure 1:**
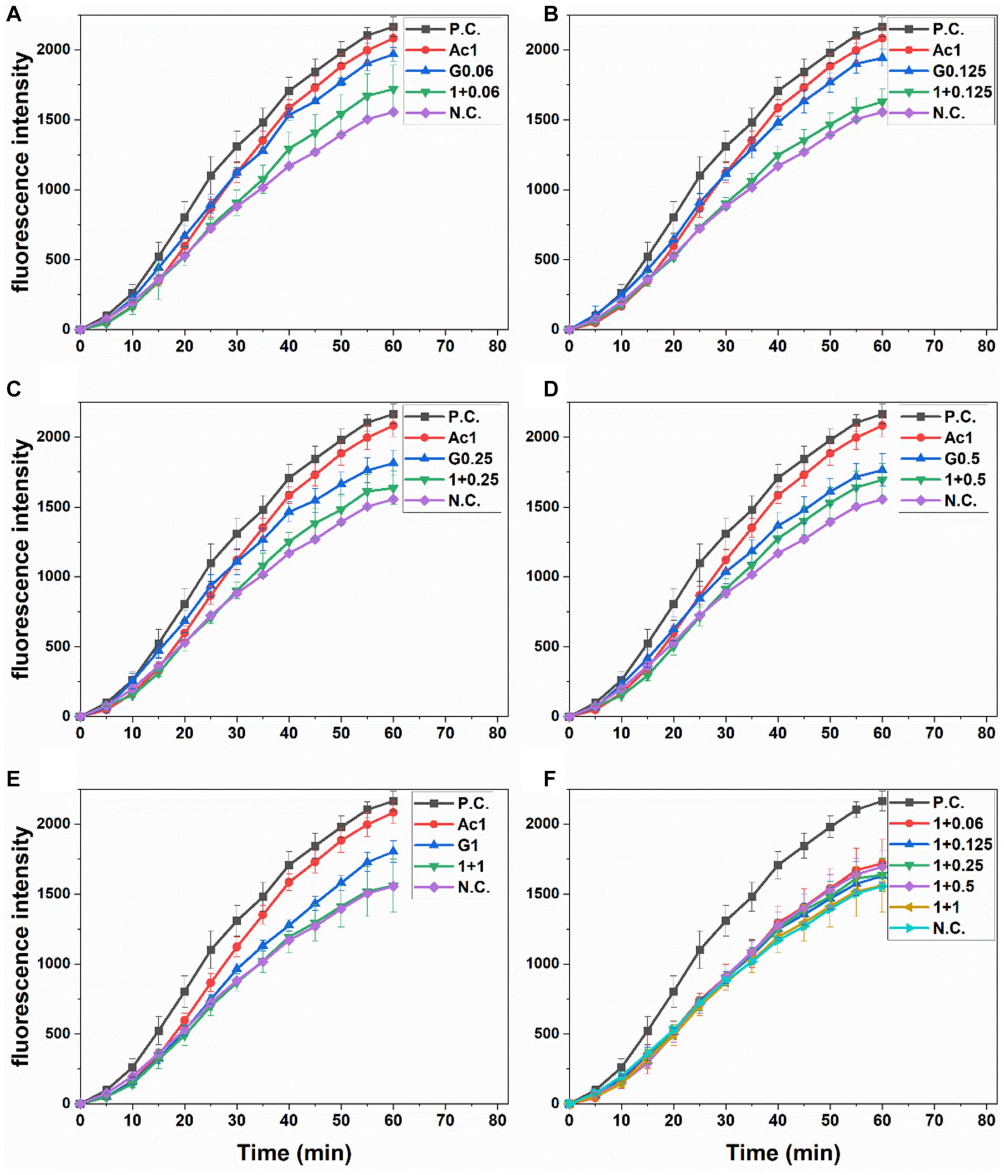
Peroxyl radical-induced oxidation of DCFH to DCF in Caco-2 cells and the inhibition of oxidation by pre-treatment with individual and combined anthocyanins and gingerols at different Ac-G combination doses (μg/mL), **(A)**: 1 + 0.06, **(B)**: 1 + 0.125, **(C)**: 1 + 0.25, **(D)**: 1 + 0.5, and **(E)**: 1 + 1, over time. **(F)**: presents combinations together. PC, positive (AAPH treated) and NC, negative (no extract nor AAPH treated) controls. All values are means ± SD.

The increased fluorescence observed in Caco-2 cells treated with the oxidant agent AAPH (positive control) compared to the negative control (only medium) is presented in Figure 1. Comparably, cells pretreated with anthocyanin or gingerol extracts showed less fluorescence than the AAPH-positive control during the incubation time. All Ac-G combined treatments showed a more reduction in fluorescence than the individual extract. The Ac-G combinations of (1 + 0.125), (1 + 0.25), and (1 + 1) μg/mL prevented the AAPH-induced oxidation in Caco-2 cells, achieving fluorescence levels closer to those of negative control cells, which were not exposed to the oxidizing agent (Figures 1B,C,E).

**Figure 2:**
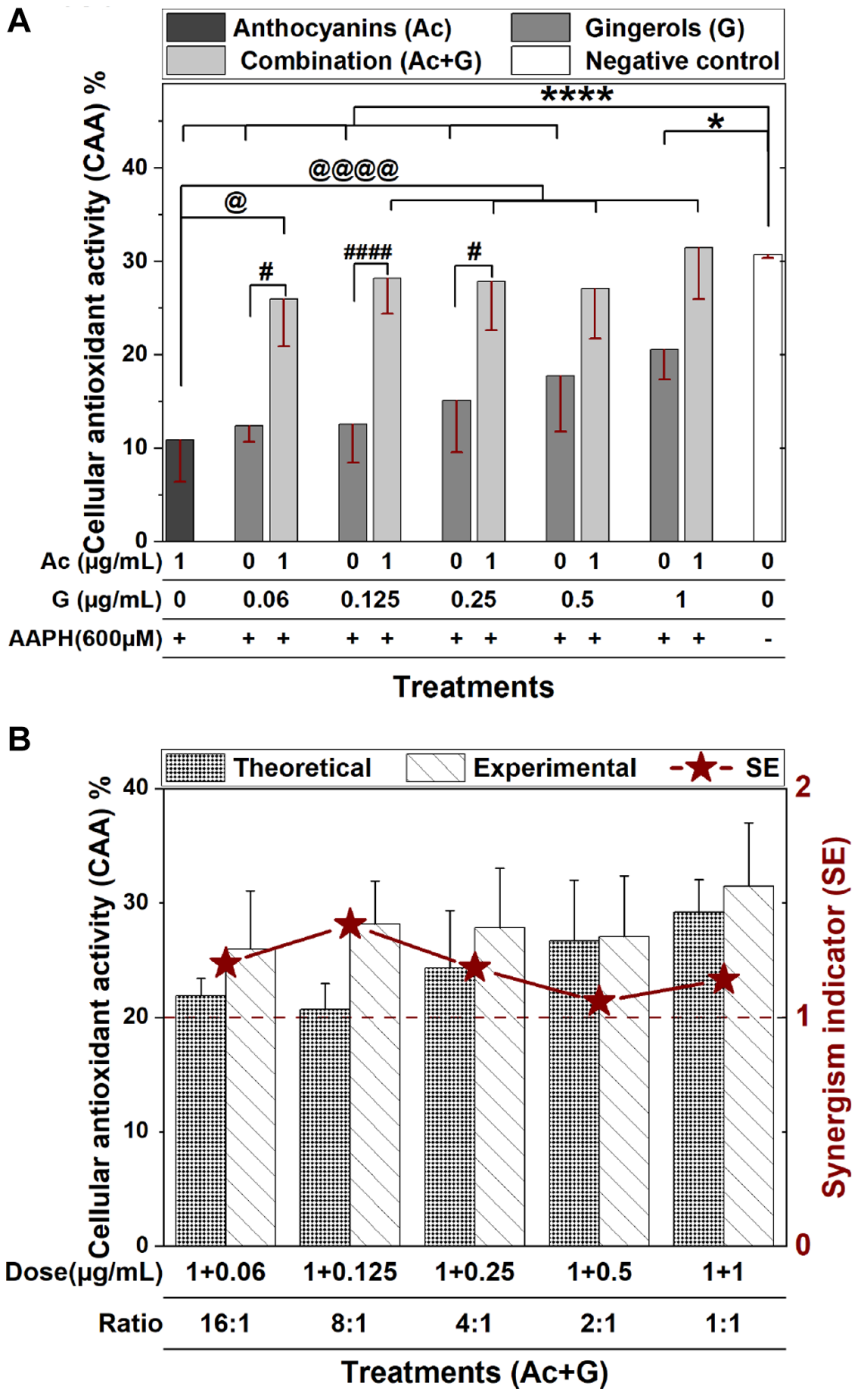
**(A)** The CAA of Caco-2 cells pretreated with individual and combined anthocyanins (Ac) and gingerols (G) compared to the control (no extract nor AAPH treatment). (* and ****), (# and ####), and (@ and @@@@) indicate significant differences compared to the control, the corresponding gingerol treatments, and the anthocyanins treatment, respectively, at (*p* < 0.05) and (*p* < 0.001), respectively. **(B)** The synergism indicator graph with a comparison of the experimental and the theoretical (calculated) CAA of Ac-G combinations.

Areas under fluorescence curves were integrated, and the CAA of each treatment was determined and presented in Figure 2A. A slight, dose-dependent increase (12.3–20.5%) was observed in the CAA of Caco-2 cells treated with gingerols at 0.06–1 μg/mL. On the other hand, the CAA of anthocyanins-treated cells (10.8%) was significantly increased (25.9–31.4%) when the anthocyanins dose was combined with those concentrations of gingerols. The increases in the CAA of Ac-G combined treatments were not dose-dependent, with all combinations showing higher levels of CAA compared to their corresponding individual treatments of gingerols; however, Ac-G combinations of (1 + 0.06) (1 + 0.125), (1 + 0.25) μg/mL (Ac/G ratios of 16:1, 8:1, and 4:1) were even significance.

The reported CAA value (10.8%) in this research for the 1 μg/mL anthocyanins treatment was comparable to the CAA values of fruit extracts (blueberry, apple, and grape) evaluated by Wolfe and Liu (22) in Hep G2 cells. However, these values were less than the CAA values (33.7–43.9%) in Caco-2 and Hep G2 cells for the bilberry and blueberry extracts reported by Bornsek et al. (37). The reported CAA values were also comparable to the CAA values of crude vegetable extracts (beetroot, red pepper, broccoli, and carrot, respectively) on Hep G2 evaluated by Song et al. (38). In this study, gingerols extract at doses of (0.06–1 μg/mL) exhibited CAA of 12.3–20.5%, which is higher than the CAA values reported by Sakulnarmrat et al. (39) for the polyphenolic-rich fraction obtained from dry ginger powder on colon adenocarcinoma (HT-29) and stomach adenocarcinoma (AGS) cells. This diversity in the CAA might be caused by different constituents in each extract and different cell lines used in each study. It has been reported that the extent of the effect of antioxidants in tissue culture-based assays depends on the types of cells and the specific antioxidants in the mixture (20).

The synergistic effect indicator (SE) for Ac-G mixtures was then calculated. SE is defined by comparing the CAA values obtained from the experiment and the expected effect values calculated from the results of both individual treatments; thus, the SE > 1, SE < 1, and SE ≅ 1 indicate synergistic, antagonistic, and additive effects, respectively. In Figure 2B, wider differences between experimental and theoretical values of the CAA were observed for the Ac-G combinations of (1 + 0.125), (1 + 0.06), and (1 + 0.25) μg/mL in favor of the experimental data. According to the synergism indicators in Figure 2B, additive to synergistic effects (1.06–1.41) (SE >1) were observed for all the tested combinations. Higher synergism indicator (SE: 1.41) was observed for the Ac-G combination of (1 + 0.125) μg/mL, followed by SE: 1.23 and 1.21 for the combinations of (1 + 0.06) and (1 + 0.25) μg/mL, reflecting the Ac/G combination ratios of 8:1, 16:1 and 4:1, respectively. Therefore, the synergistic effect opportunity is more prominent at the lower gingerols amount in the mixture.

### 3.3. Effects of Ac-G combined pre-treatments on the cytoprotective effects

The protective effects of anthocyanins and gingerols and their combinations against oxidative stress were further evaluated by measuring the cell viability of pretreated Caco-2 cells after a 24 h exposure to oxidative stress induced by 350 μM t-BHP.

**Figure 3:**
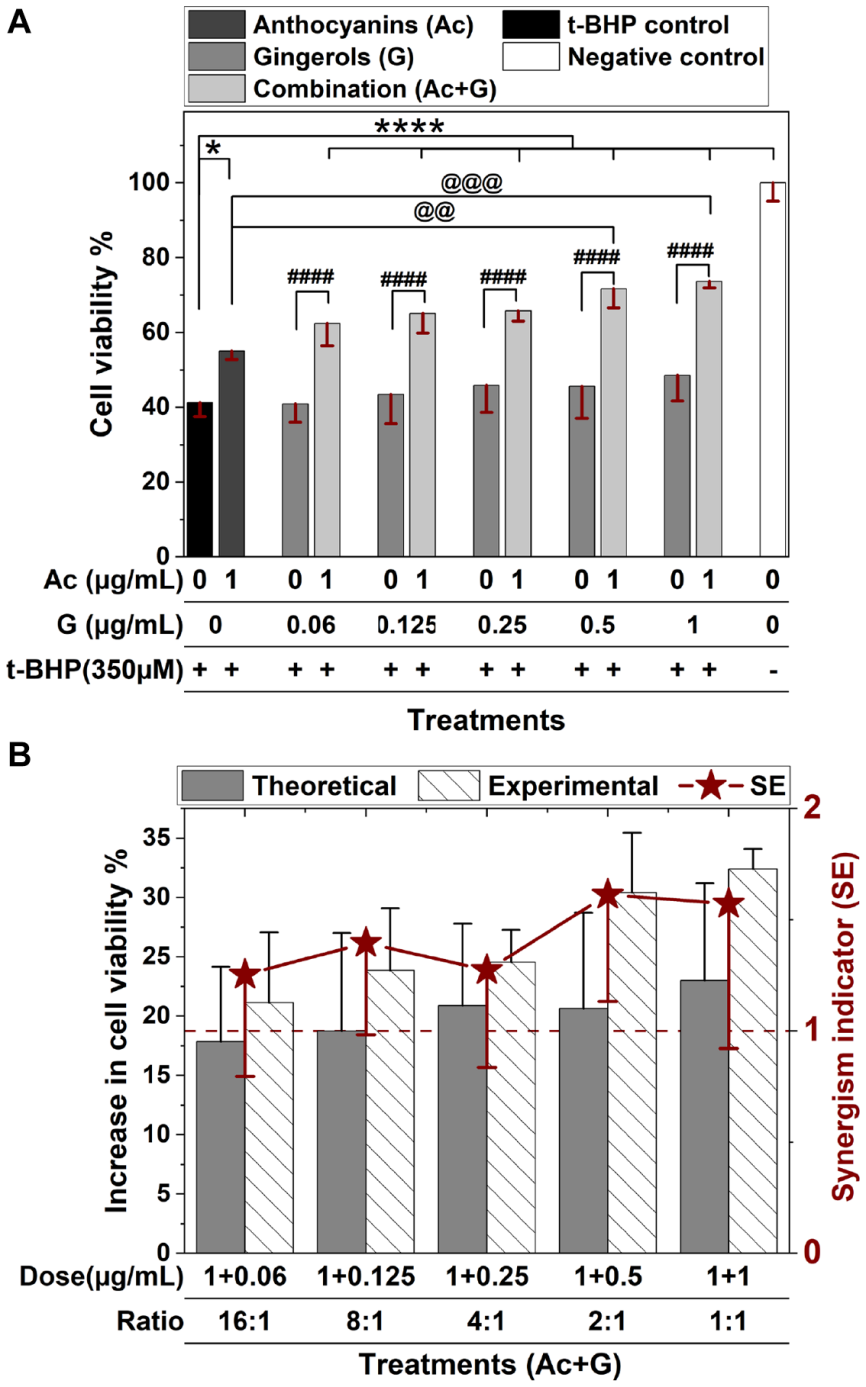
Protective effects of anthocyanins (Ac), gingerols (G), and their combined treatments against 24 h t-BHP induced oxidative stress on Caco-2 cell viability. Values were expressed as mean ± SD. **(A)** Cell viability of extract pre-treated cells after t-BHP treatment. * and ****: Indicate significant differences compared to the control, at *p* < 0.05 and *p* < 0.001, respectively. ####: Indicates significant differences compared to the corresponding gingerol treatments at *p* < 0.001. @@ and @@@: Indicate significant differences compared to the anthocyanins’ treatment, at *p* < 0.01 and *p* < 0.005, respectively. **(B)** The synergism indicator (SE) graph with a comparison of the experimental and the theoretical (calculated) increase in the cell viability when cells were pretreated with Ac-G combinations.

In Figure 3A, treating cells with t-BHP resulted in a 60% reduction in cell viability. Cells pretreated with 1 μg/mL anthocyanins showed a significant (*p < 0.05*) increase in cell viability (55.1%) compared to the control (41.2%), while gingerol treatments (0.125–1 μg/mL) exhibited a slight dose-dependent increase in cell viability (43.3–48.5%). Meanwhile, cells pretreated with combined anthocyanin and gingerol extracts showed a significant (*p* < 0.001) dose-dependent increase in cell viability (62.4–73.6%) compared to the control and the corresponding gingerol treatments. Compared to the anthocyanins’ treatment, only Ac-G combinations of (1 + 0.5) and (1 + 1) μg/mL exhibited significant increases at (*p* < 0.01) and (*p* < 0.005), respectively.

The synergistic effect indicator (SE) for Ac-G combinations was also calculated for the cytoprotective activity after 24 h exposure to the t-BHP-induced oxidative stress. Synergistic effects were observed for all the tested combinations, with SE values in the range of 1.25–1.61 (SE > 1) (Figure 3B). The Ac-G combinations with higher doses of gingerols (1 + 0.5) and (1 + 1) μg/mL (Ac/G ratios of 2:1 and 1:1) exhibited higher levels of synergism (SE: 1.61 and 1.57), respectively. These data indicate that combining anthocyanins and gingerols protected against oxidative stress revealed in both CAA and cytoprotective assays to maintain cell viability in Caco-2 cells.

### 3.4. Effects of Ac-G combined pre-treatments on the cellular antioxidant defense network response against oxidative stress

During oxidative stress, the endogenous antioxidative system (antioxidant enzymes and GSH) is overwhelmed by high free radicals and ROS levels, leading to oxidative damage to cellular structures, including membrane lipids (32). This study explored the effects of combined and individual anthocyanins and gingerols pre-treatments in supporting the cellular response to induced oxidative stress by evaluating GSH content, GPx and SOD enzyme activities, and lipid peroxidation levels.

#### 3.4.1. Reduced glutathione content under stressed conditions

**Figure 4:**
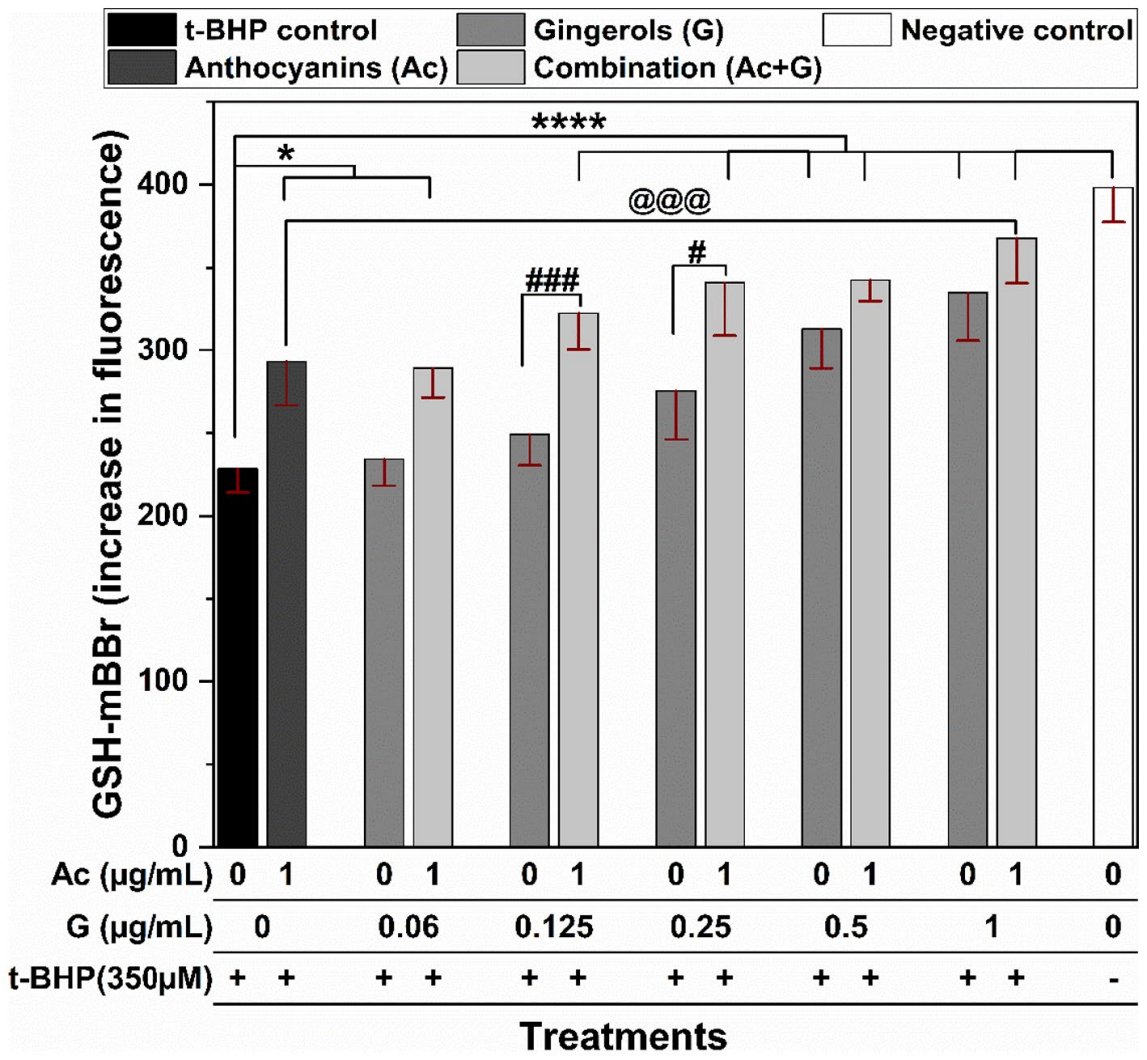
The effects of anthocyanins (Ac) and gingerols (G) treatments individually and in combination on the intracellular GSH content in Caco-2 cells after 3 h t-BHP-induced oxidative stress on the pretreated cells. * and **** indicate significant differences compared to the t-BHP control at (*p* < 0.05) and (*p* < 0.001), respectively. # and ### indicate significant differences compared to the corresponding treatment of gingerols at (*p* < 0.05) and (*p* < 0.005), respectively. @@@ indicates a significant difference compared to the anthocyanins’ treatment at (*p* < 0.005).

Cells exposed to oxidative stress induced by 3 h treatment with t-BHP (t-BHP control) exhibited a significant (*p* < 0.005) reduction in the GSH levels when compared to the negative control (non-treated cells) (Figure 4). Combined treatments of 1 μg/mL anthocyanins with (0.125–1 μg/mL) gingerols significantly (*p* < 0.005) prevented the reduction in GSH levels. Only the (1 + 1) Ac-G combination showed significantly (*p* < 0.005) higher GSH levels compared to the individual anthocyanins, but this was not significant (*p* < 0.05) compared to the gingerols treatment alone. Meanwhile, only Ac-G combined doses of (1 + 0.125) and (1 + 0.25) μg/mL showed significantly higher GSH levels than the corresponding gingerols alone at (*p* < 0.005) and (*p* < 0.05), respectively. In addition, more differences between individual and combined treatments were observed at the Ac-G combinations of (1 + 0.125) and (1 + 0.25) μg/mL, which might contribute to the higher synergism indicator values obtained in the CAA at these combinations.

#### 3.4.2. Antioxidant enzymes

Antioxidant enzymes, including SODs, and GPxs, work cooperatively and play an essential role in redox homeostasis, and they are distinct in their cellular location. Both enzymes are present in the cytoplasm and mitochondrial matrix in different forms. Each form uniquely regulates cellular signaling pathways and protects cells from oxidative damage. SOD enzymes are the first defense line by converting superoxide radicals to H_2_O_2_ and oxygen. GPxs help eliminate intracellular H_2_O_2_ and reduce organic peroxides, such as fatty acid hydroperoxides, by coupling their reduction with the oxidation of GSH and transforming H_2_O_2_ into O_2_ and H_2_O (40, 41). Inducing the activity of antioxidant enzymes is one of the critical mechanisms to defend against oxidative stress conditions in the cells; however, excessive oxidative stress may inactivate those enzymes (4).

**Figure 5:**
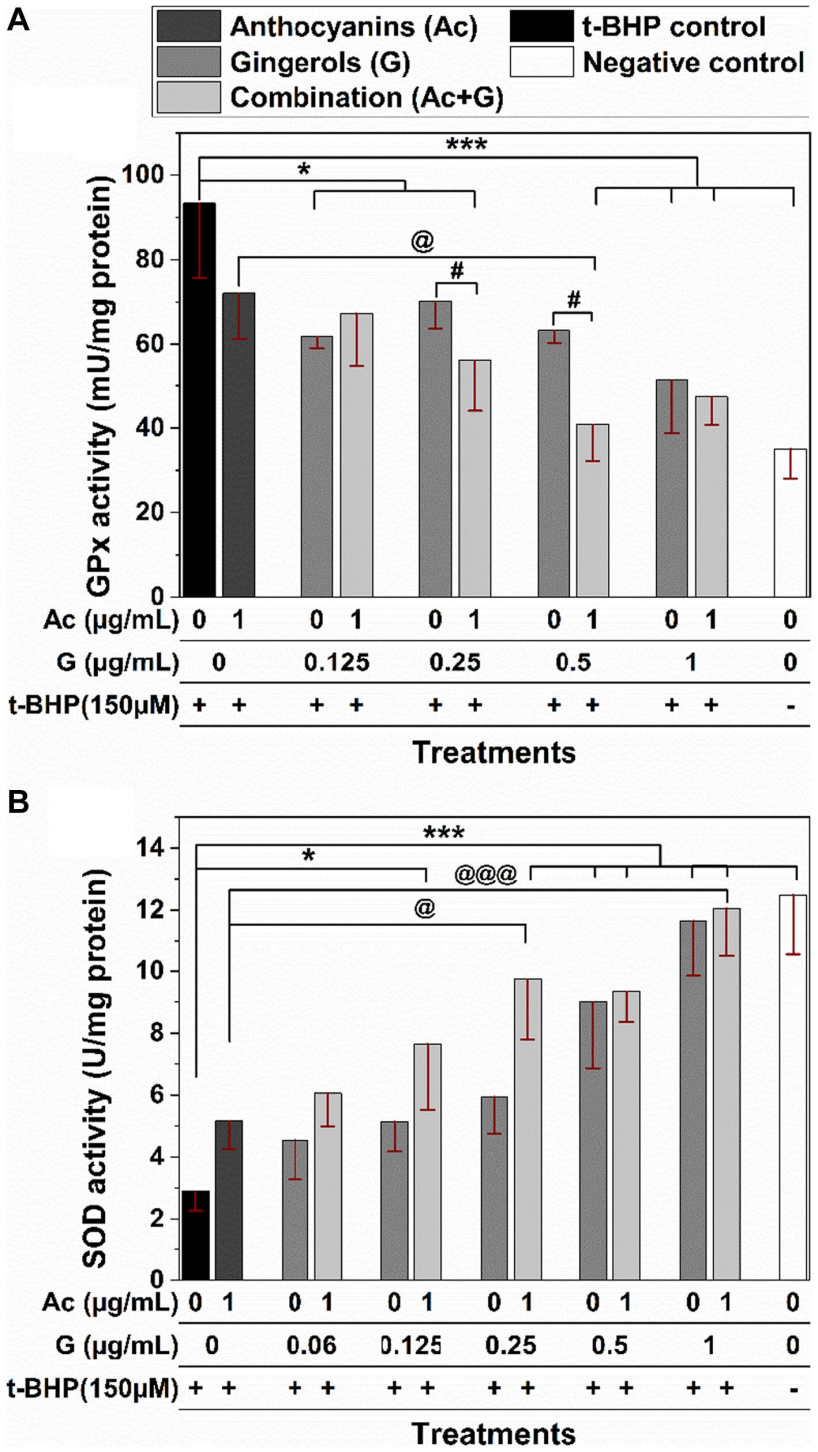
Effects of anthocyanins (Ac) and gingerols (G) pre-treatments, individually and in combination, on the antioxidant enzyme activity of GPx **(A)** and SOD **(B)** enzymes against 24 h t-BHP-induced oxidative stress in Caco-2 cells. (* and ***) and (@ and @@@) indicate significant differences compared to the t-BHP control and anthocyanins’ treatment at (*p* < 0.05 and *p* < 0.005), respectively; # indicates significant differences compared to the corresponding gingerol treatments at *p* < 0.05.

In Figure 5A, exposing Caco-2 cells to 150 μM t-BHP for 24 h caused a 2-fold increase in the activity of the GPx enzyme (t-BHP positive control). The induced increase in GPx activity was significantly prevented when cells were pretreated with Ac-G combinations of (1 + 0.5) and (1 + 1) μg/mL at (*p* < 0.005) and (1 + 0.25) μg/mL at (*p* < 0.05). Two Ac-G combined treatments, (1 + 0.5) and (1 + 0.25) μg/mL, significantly (*p* < 0.05) lowered the induced GPx activity compared to the corresponding gingerols treatment alone. Meanwhile, only the Ac-G combination of (1 + 0.5) μg/mL exhibited significantly (*p* < 0.05) lower activity levels than the anthocyanins treatment alone. It showed similar levels to the negative control (cells without treatment), indicating the synergistic effect at such a combined dose. It might contribute to the higher synergism levels observed for this combination in the cytoprotective effect.

Treating Caco-2 cells with t-BHP caused a 4-fold decrease in the activity of the SOD enzyme compared to the negative control (Figure 5B). A lower reduction in the SOD enzyme activity was observed when cells were pretreated with anthocyanins, gingerols, and their combinations, indicating their effectiveness in protecting the enzyme molecules from oxidation. The SOD activity was significantly higher for the cells pretreated with the Ac-G combined dosages of (1 + 0.25), (1 + 0.5), and (1 + 1) μg/mL at (*p* < 0.005), and for the (1 + 0.125) μg/mL at (*p* < 0.05) when compared to the t-BHP control. Compared to the anthocyanins’ treatment, only Ac-G combined dosages of (1 + 0.25) and (1 + 1) μg/mL showed significance at (*p* < 0.05) and (*p* < 0.005), respectively. Moreover, wider differences were observed for the Ac-G combined treatments of (1 + 0.25) and (1 + 0.125) μg/mL compared to the corresponding gingerol treatments. The higher effects of these Ac-G combinations in preventing SOD inactivation can be attributed to the synergism between anthocyanins and gingerols at these combination doses in their CAA.

Even though many phenolic compounds contribute to the antioxidant defense mechanisms in the cells, their beneficial effects are still dependent on the type and source of the actual oxidative stress (4, 42). The t-BHP is stable in aqueous solutions and induces a constant cellular stress (4), which gives a long-sustained oxidative effect on the cells. In addition, t-BHP is a hydroperoxide, and GPx catalyzes H_2_O_2_ and organic hydroperoxides (43). Thus, the presence of t-BHP caused hydroperoxide oxidative stress and increased the activity of the GPx enzyme, and reversed GPx activity means an antioxidant effect. Furthermore, the increased GPx activity under oxidative stress (Figure 5A) was also associated with decreased GSH levels (Figure 4). It is known that GPx relies on GSH (specific co-factor) for the necessary reducing equivalents of H_2_O_2_ (43). On the other hand, the substantial reduction in the SOD activity observed in the stressed cells can be attributed to the inactivation of the enzyme molecules in the cytoplasm induced by the oxidant t-BHP, as SOD enzymes work on the superoxide radicals, not hydroperoxides.

The regulation of antioxidant enzymes depends on many factors, including organ specificity, age, and the availability of active site cofactors (40). Therefore, the activity and expression of antioxidant enzymes and GSH levels are organ-specific and could also be modulated by the metabolic requirements of the tissue (41). Moreover, the response of the antioxidant defense enzymes may depend on the experimental model and conditions utilized in each specific study. For example, in Alía et al. (44) study, a 3 h treatment of 200 μM t-BHP induced significant increases in the GPx and SOD enzyme activities in Hep G2 cells. Meanwhile, Bamdad et al. (34) reported decreases in SOD and GPx activities in Chang liver cells stressed with 150 μM t-BHP for 24-h. In this research, a 2-fold increase in GPx activity was observed in Caco-2 cells, accompanied by a dramatic decrease in the SOD activity in the 24 h t-BHP-stressed Caco-2 cells compared to the unstressed ones. Furthermore, pre-treatment of Caco-2 cells with the Ac-G combinations significantly changed the antioxidant enzymes GPx and SOD activities, bringing their levels comparable to those of negative controls (non-treated cells) at specific combination dosages. These results further support the synergism potential of anthocyanins and gingerols in their antioxidant effects.

#### 3.4.3. Lipid peroxidation

**Figure 6:**
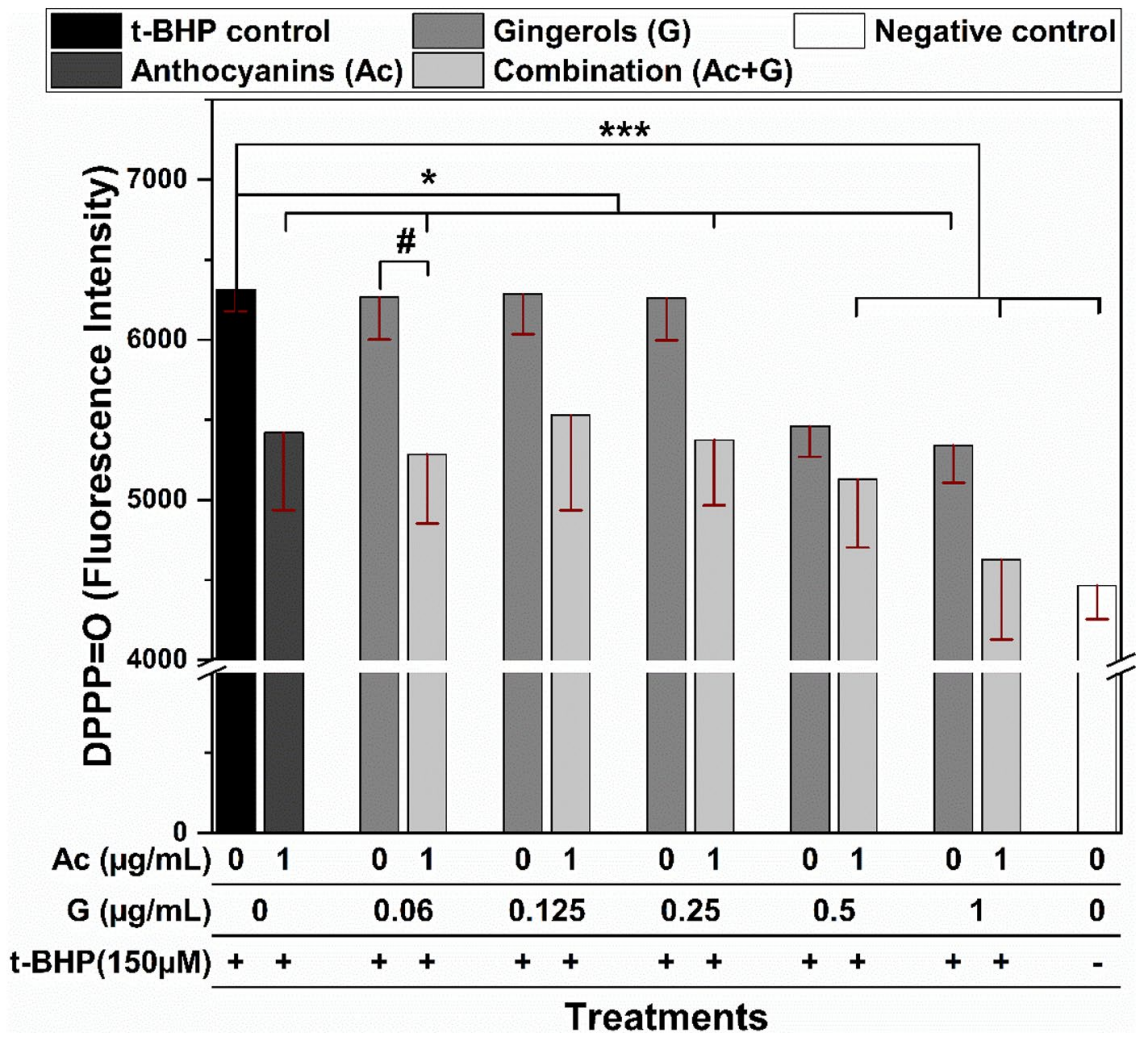
Fluorescence intensity of the DPPP-labeled Caco-2 cells pretreated with anthocyanins (Ac), gingerols (G), and the combined (Ac + G) treatments after oxidation induced by t-BHP. * and ***: indicate significant differences compared to the t-BHP control at *p* < 0.05 and *p* < 0.005, respectively; #: indicates significant differences compared to the corresponding gingerols treatment at *p* < 0.05.

The effect of the anthocyanin and gingerol combinations to prevent lipid peroxidation was evaluated when Caco-2 cells pretreated with extract were stressed by t-BHP. DPPP is a non-fluorescent molecule known to react with hydroperoxide within the cell membranes to produce diphenyl-1-pyrenylphosphine oxide (DPPP=O) that is fluorescent (35). In Figure 6, t-BHP-treated cells showed a significant (*p* < 0.005) increase in the fluorescent intensity compared to the non-treated cells (negative control). Comparably, almost all Ac-G combined pre-treatments significantly lowered the fluorescent intensity in cells under t-BHP oxidative stress. Further, the reduction in fluorescent intensity for the two Ac-G combinations of (1 + 0.5) and (1 + 1) μg/mL was more significant (*p* < 0.005). However, a significant difference (*p* < 0.05) was observed only for the Ac-G combination of (1 + 0.06) μg/mL compared to the corresponding individual gingerol treatments.

The inhibition of lipid peroxidation in the stressed Caco-2 cells by Ac-G combinations could also be related to the effects of these treatments in increasing the GSH levels and preserving the SOD enzyme. Moreover, the positive correlation between the inhibition of cellular lipid peroxidation (Figure 6) and the reduction in the GPx activities (Figure 5A) of those cells could support the role of these treatments in scavenging the oxidants, where reduced ROS levels were observed under all those combined treatments.

All these results indicate higher effects of Ac-G combinations in supporting the endogenous system and reducing the impacts of the induced oxidative stress. To explore the effects of different treatments on the cells under normal conditions, before being exposed to oxidative stress, the ROS generation and GSH content were evaluated in cells treated with combined and individual anthocyanins and gingerols.

### 3.5. Effects of Ac-G combined pre-treatments on the cellular ROS production and GSH level under normal conditions

#### 3.5.1. ROS generation

**Figure 7:**
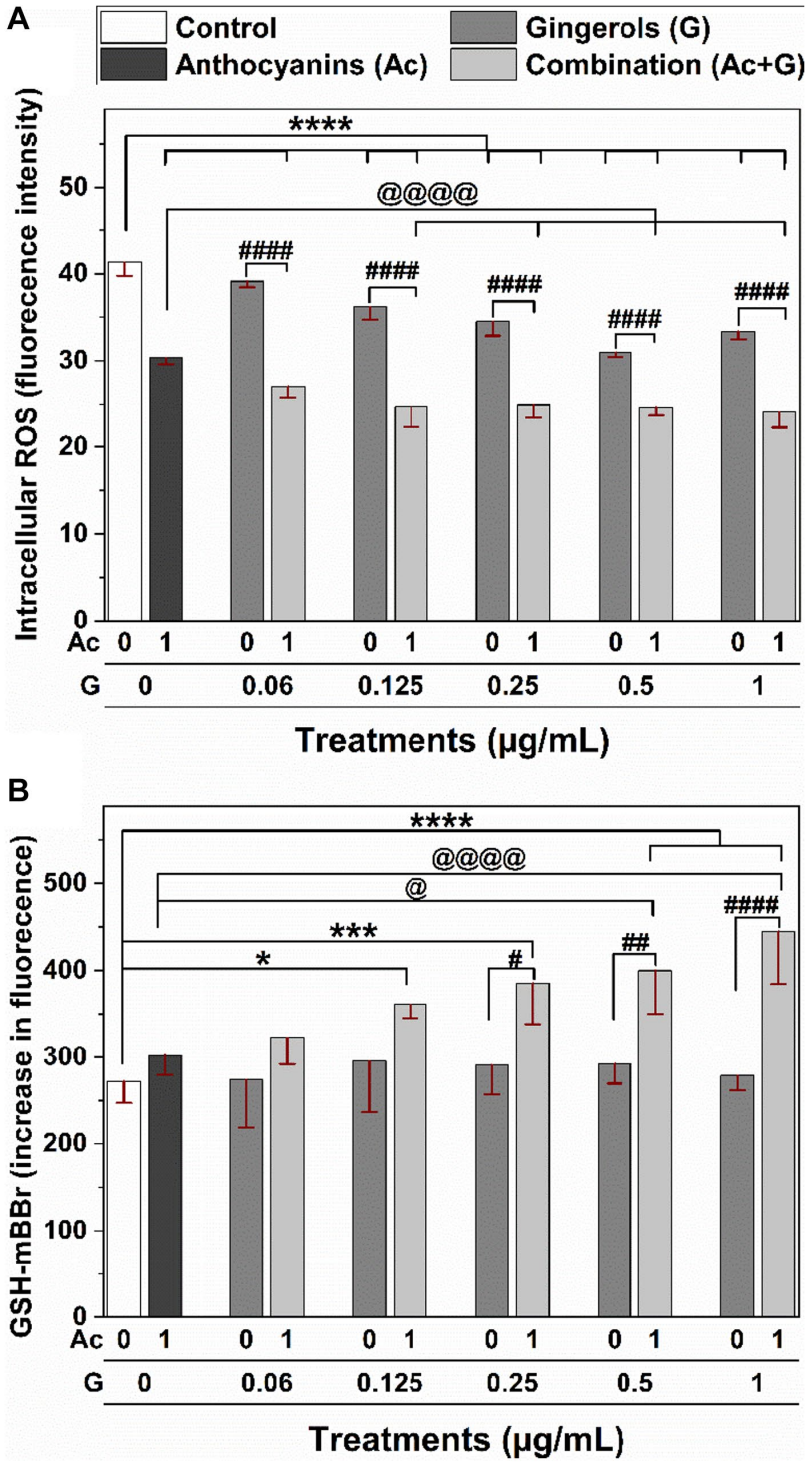
Effects of individual and combined anthocyanins (Ac) and gingerols (G) treatments on the intracellular **(A)** ROS generation and **(B)** GSH content in Caco-2 cells. *, *** and **** indicate significant differences compared to the control at (*p* < 0.05), (*p* < 0.005), and (*p* < 0.001), respectively. #, ##, and #### indicate significant differences compared to the corresponding treatment of gingerols at (*p* < 0.05), (*p* < 0.01), and (*p* < 0.001), respectively. @ and @@@@ indicate significant differences compared to the anthocyanins’ treatment at (*p* < 0.05) and (*p* < 0.001), respectively.

Individual and combined treatments showed a significant (*p* < 0.001) reduction in ROS generation compared to the non-treated cells as a control (Figure 7A). Compared to the individual extracts, Ac-G combinations of (1 + 0.125) – (1 + 1) μg/mL (Ac/G ratios of 8:1–1:1) significantly (*p* < 0.001) reduced ROS levels. However, Ac-G combinations of (1 + 0.125) and (1 + 0.25) μg/mL (Ac/G ratios of 8:1 and 4:1) showed greater reduction compared to each extract alone and other combinations, which might contribute to the higher synergism levels observed in the CAA activity (Figure 2B) at those combinations. The kit used in this assay detects intracellular ROS in the superoxide and hydroxyl radical forms. Thus, the results reflect the effects of the extracts on scavenging endogenous superoxide and hydroxyl radicals in the cells.

#### 3.5.2. Reduced glutathione content under normal conditions

The effects of anthocyanins, gingerols, and their combinations on the intracellular GSH levels of Caco-2 cells were measured under normal physiological conditions (no induced stress) (Figure 7B). No significant changes were observed in the cells treated with individual anthocyanins and gingerols at 1 and 0.06–1 μg/mL, respectively. However, significant (*p* < 0.05) increases in the GSH levels compared to the control (untreated cells) were observed when cells were treated with the Ac-G combinations. Particularly, the increases in the GSH levels for the Ac-G combined treatments (1 + 0.5) and (1 + 1) μg/mL showed more significance (*p* < 0.001) compared to the control. Moreover, they significantly differed from the corresponding gingerol treatments and the anthocyanins.

It was also observed that treating the cells with gingerols alone did not affect the levels of GSH (Figure 7B) but reduced the ROS levels (Figure 7A) in a dose-dependent manner. Meanwhile, when those doses of gingerols were combined with anthocyanins, the increase in GSH levels was significant and dose-dependent. However, the reduction in the ROS levels was not dose-dependent, although significant. These results suggest that the synergistic effects applied by combined treatments are more toward lower doses in reducing the ROS generation may contribute to the higher synergism in the CAA at those treatments. Meanwhile, the synergistic effects applied by combined treatments are more toward higher doses in increasing the GSH levels, which might contribute to the higher synergism in the cytoprotective results for those treatments. These results suggest some of the mechanisms involved in the synergistic effects that helped the cells be better prepared against oxidative stress when oxidant agents were applied in the CAA and the cytoprotective assays.

### 3.6. Discussion of the synergistic effect

Gingerol treatments significantly increased the CAA in a dose-dependent manner (Figure 2A), while anthocyanin treatment significantly increased the cell viability of stressed cells (Figure 3A). The combination of anthocyanins and gingerols provided cumulative and additive effects and evoked an effect defined as synergism. Results of Ac-G combinations with lower gingerols concentrations, such as an 8:1 ratio (dose of 1 + 0.125 μg/mL), showed a greater effect in the short term by increasing the CAA levels and reducing the induced ROS levels. Ac-G combinations with higher gingerol concentrations, such as a 2:1 ratio (dose of 1 + 0.5 μg/mL), showed higher impacts in preventing the damaging effects of 24 h oxidative stress, as observed in the cytoprotective data (Figure 3A). These together suggest that various combination ratios activate different mechanisms in each stage.

Synergism of Ac-G combined treatments in the CAA was higher at Ac-G combination ratios of 8:1, 16:1, and 4:1 [(1 + 0.125), (1 + 0.06), and (1 + 0.25) μg/mL], which also showed greater impact in reducing ROS levels than the individual gingerol treatments. It is suggested that the synergistic effects of the Ac-G combined treatments in CAA result from their impact on protecting the SOD enzyme and reducing the oxidative stress-induced depletion in cellular GSH levels, where the two Ac-G combination ratios of 8:1 and 4:1 [(1 + 0.125) and (1 + 0.25) μg/mL] were significantly more effective in preserving the SOD enzyme activity and reducing GSH depletion when compared to the corresponding gingerol treatments.

In the cytoprotective effects, higher levels of synergism were observed for the Ac-G combined doses of (1 + 0.5) and (1 + 1) μg/mL (Ac-G ratios of 2:1 and 1:1) (Figure 3A). Treating cells with these two combination dosages significantly increased the levels of cellular GSH compared to the control and corresponding individual treatments (Figure 7B) and showed significant prevention of the t-BHP-induced increase in GPx activity (Figure 5A). Notably, the Ac-G combinations of (1 + 0.5) μg/mL (Ac/G ratio of 2:1) could bring the GPx activity levels comparable to that of the negative control.

Results indicate that lower gingerols in combination affect more in protecting cell components against oxidative stress. At the same time, more anthocyanins increase the readiness of the cell before exposure to stress by increasing GSH levels.

Combining more than one agent impacts multiple pathways and may provide cumulative or additive effects to evoke synergism. For example, anthocyanins treatment alone significantly reduced ROS generation (Figure 7A) and prevented the induced GSH depletion (Figure 4) in the cell. Meanwhile, gingerols treatments alone induced a significant dose-dependent reduction in ROS generation (Figure 7A), prevented GSH depletion (Figure 4), and caused an increase in SOD activity (Figure 5B). However, the effect of the combination was even greater than each alone. Thus, anthocyanins and gingerols combinations targeted more pathways and induced more effects than each alone, explaining the synergistic effect observed in this study.

The GSH plays an essential role in maintaining the intracellular redox status, and cellular GSH levels can be depleted when exposed to oxidative stress. Therefore, intervention with exogenous antioxidants is required to support the endogenous antioxidant system in protecting the cells from oxidation and restoring the normal redox state. In this study, treating cells with individual anthocyanins or gingerols slightly changed the GSH levels (Figure 7A), but they made the cells more tolerant and reduced the depletion of the cellular GSH level in the presence of oxidative stress (Figure 4). Meanwhile, Ac-G combinations increased GSH levels in non-stressed cells (Figure 7A) and much better maintained the GSH levels under induced oxidative stress (Figure 4). The GSH values were comparable to the GSH levels in the control cells (non-stressed cells). The significant increases in the GSH levels in the non-stressed cells (Figure 7B) represent the Ac-G combination effects in increasing the GSH levels even before exposure to oxidative stress. Such significant increases in the GSH level observed at these Ac-G combinations may contribute to the higher synergism in the cellular response to maintain the redox status and protect the cells against induced oxidative stress.

Anthocyanin synergy was previously reported when combining raspberries with adzuki bean extracts and with sumac extracts using chemical-based assays (21, 45). In addition, positive interactions were reported between anthocyanins (cyanidin-3-glucoside, malvidin- 3-glucoside, or pelargonidin-3-glucoside) and quercetin in FRAP assay, but not in DPPH assay (46). As far as known, this study is the first to demonstrate gingerols’ synergy with anthocyanins to induce antioxidant activity in cell models.

The low bioavailability of anthocyanins and gingerols limits their health benefits. Thus, achieving the same biological effects at a lower dosage by combining these two phenolic compounds may provide a new strategy to overcome the common bioavailability issue. The Ac-G combination at the doses of (1 + 0.125), (1 + 0.25), and (1 + 0.5) μg/mL could be considered for new natural health products and functional food development with enhanced antioxidant effects to prevent oxidative stress and associated chronic health conditions.

Functional proteins play a significant role in cell activities; therefore, analyzing the expression of such cellular proteins provides crucial insights into studying cellular heterogeneities. For cost and ethical reasons, this work focused on studying potential synergism in cellular modules, and some involved biomarkers were explored. For future studies, protein regulators such as transcription factors, which are more likely to be involved in the synergistic interaction, should be evaluated using cell models. For example, transcription factors Nrf2/Keap1 (nuclear factor-E2-related factor 2/Kelch-like ECH-associated protein 1), NF-kB, p53, and activator protein-1 (AP-1) have been reported to be regulated by and in response to oxidative stress, and they were linked to cell death *via* complex signaling pathways (47, 48). The positive results in cell models will justify studying the synergistic effect of anthocyanins and gingerols and their effective dosage in animal and clinical studies in the future.

## 4. Conclusion

In conclusion, the Ac-G mixtures (1 + 0.06) – (1 + 1) μg/mL showed synergistic responses in CAA and cytoprotective activities in Caco-2 cells with SE values up to 1.61. The highest SE value observed in the CAA was 1.41 at the Ac-G combined dose of (1 + 0.125) μg/mL, while the highest SE value observed in the cytoprotective effects was 1.61 at the Ac-G combined dose of (1 + 0.5) μg/mL. Furthermore, Ac-G combinations protected the cellular redox status against induced oxidative stress by increasing GSH content. They positively affected the cellular enzymatic antioxidant defense system by reducing the induced GPx enzyme activity and saving SOD enzyme activity. Moreover, Ac-G combinations significantly reduced cellular ROS generation. They increased GSH levels under physiological conditions, which helped the cells be better prepared against oxidative stress when exposed. Thus, Ac-G combinations could support the cellular antioxidant defense systems at different levels.

Therefore, the synergy derived from Ac-G formulations in their antioxidant and cytoprotecting effects is a promising health option for people, health professionals, and the food industry. Introducing those antioxidants together through dietary or supplementary sources could support the endogenous system to protect the cells from oxidation. In this study, biomarkers associated with the antioxidant activity and redox pro-survival pathways have been investigated in cell-based assays, which could help better guide the supplementary usage of such a combination; however, the metabolism of antioxidants synergy should be evaluated *in vivo*. Thus, additional research on animal models is required to study the synergistic effects between anthocyanins and gingerols in inducing the antioxidant capacity, which may provide reasonable evidence for further clinical testing.

## Data availability statement

The original contributions presented in the study are included in the article/Supplementary material, further inquiries can be directed to the corresponding author.

## Ethics statement

Ethical approval was not required for the studies on humans in accordance with the local legislation and institutional requirements because only commercially available established cell lines were used.

## Author contributions

All authors contributed to the study’s conception and data interpretation. AA was responsible for the conceptualization, experimental design, performing the experiments, data analysis, visualization, and interpretation, and writing the first draft of the manuscript. VM assisted with the data analysis, illustration and interpretation, and revisions to the manuscript. LC is the corresponding author responsible for the experimental design, resources, supervision, revisions, and manuscript submission. All authors contributed to the article and approved the submitted version.

## Conflict of interest

The authors declare that the research was conducted in the absence of any commercial or financial relationships that could be construed as a potential conflict of interest.

## Publisher’s note

All claims expressed in this article are solely those of the authors and do not necessarily represent those of their affiliated organizations, or those of the publisher, the editors and the reviewers. Any product that may be evaluated in this article, or claim that may be made by its manufacturer, is not guaranteed or endorsed by the publisher.

## Abbreviations

Ac, Anthocyanins
CAA, Cellular antioxidant activity
G, Gingerols
ROS, Reactive oxygen species
SE, Synergistic effect indicator
