# Supplementary material for: Gingerols synergize with anthocyanins to induce antioxidant activity in vitro
Source: Front Nutr. 2023 Sep 8;10:1229015. doi: 10.3389/fnut.2023.1229015 (PMC10514514; doi:10.3389/fnut.2023.1229015)
Supplement: Supplementary file 1 [file Data_Sheet_1.docx]

Supplementary Material

Gingerols synergize with anthocyanins to induce antioxidant activity *in vitro*

**Amna E. Abdurrahim^1,2^,** **Vera C. Mazurak^1^,** **Lingyun Chen^1^**^*^

^1^Department of Agricultural, Food & Nutritional Science, University of Alberta, Edmonton, Alberta, Canada

^2^Department of Food & Nutritional Science, College of Medical Technology-Misurata, Misurata, Libya

*Corresponding Author

Dr. Chen,

Tel.: +1-780-492-0038; Fax: +1-780-492-8914.

Email address: [lingyun.chen@ualberta.ca](mailto:lingyun.chen@ualberta.ca)





**Supplementary Information 1**. HPLC graphs of (A) anthocyanins-rich bilberry extract (Ac) with anthocyanin standards and (B) ginger extract (G) with gingerol standards.

**Supplementary Information 2**. Soluble fractions of bilberry and ginger extracts and the total amounts of anthocyanins and gingerols were detected by HPLC in water solutions.

| **Sample Extract** | **Soluble Fraction (mean± SD)**  **(mg/1 mg extract)** | **HPLC (mean± SD)**  **(mg/1 mg extract)** | **Active constituents (HPLC)/soluble fraction %** |
| --- | --- | --- | --- |
| Bilberry extract in water | 0.80 ± 0.081 | 0.78 ± 0.027 | 97 |
|  |  |  |  |
| Ginger extract in water | 0.166 ± 0.057 | 0.106 ± 0.013 | 63.9 |

**Supplementary Information 3**. Amounts of different anthocyanins detected by HPLC in the rich bilberry extract (Ac), quantified as mg Cyanidin 3-O-glucoside equivalent/mg extract.

| **Peak’s number**  **(Supporting Information 1-A)** | **Anthocyanin’s name** | Amounts of anthocyanins  **(mg****/1mg extract)** |
| --- | --- | --- |
| 1 | Delphinidin 3-O-galactoside | 0.1177168 |
| 2 | Delphinidin 3-O-glucoside | 0.11076859 |
| 3 | Cyanidin 3-O-galactoside | 0.08059555 |
| 4 | Delphinidin 3-O-arabinoside | 0.0773615 |
| 5 | Cyanidin 3-O-glucoside | 0.10518177 |
| 6 | Petunidin 3-O-galactoside | 0.06532282 |
| 7 | Cyanidin 3-O-arabinoside | 0.05302851 |
| 8&9 | Petunidin 3-O-glucoside  Peonidin 3-O-galactoside | 0.07947637 |
| 10 | Petunidin 3-O-arabinoside | 0.02955589 |
| 11 | Peonidin 3-O-glucoside | 0.04153463 |
| 12 | Malvidin 3-O-galactoside | 0.04992446 |
| 13 | Peonidin 3-O-arabinoside | 0.04466094 |
| 14 | Malvidin 3-O-glucoside | 0.08191626 |
| 15 | Malvidin 3-O-arabinoside | 0.0362396 |

**Supplementary Information 4**. Amounts of different gingerols detected by HPLC in the ginger root extract (G), quantified as mg 6-gingerol equivalent/mg extract.

| **Peak’s number**  **(Supporting Information 1-B)** | **Gingerol’s name** | Amounts of gingerols  **(mg/1mg extract)** |
| --- | --- | --- |
| 1 | 6-gingerol | 0.06543813 |
| 2 | 8-gingerol | 0.01361628 |
| 3 | 6-shogaol | 0.01441345 |
| 4 | 10-gingerol | 0.01218579 |
